# Characterization of the complete plastome of *Ophrys aveyronensis*, a Euro-Mediterranean orchid with an intriguing disjunct geographic distribution

**DOI:** 10.1080/23802359.2019.1670748

**Published:** 2019-09-26

**Authors:** Joris A. M. Bertrand, Anaïs Gibert, Christel Llauro, Olivier Panaud

**Affiliations:** a Laboratoire Génome & Développement des Plantes (UMR 5096 UPVD/CNRS), University of Perpignan Via Domitia, Perpignan, France;; b PSL Université Paris, EPHE-UPVD-CNRS, USR 3278 CRIOBE, Université de Perpignan, Perpignan, France

**Keywords:** Ophrys sphegodes aveyronensis, Bee orchid, Orchidaceae, chloroplast (cp) genome, illumina sequencing

## Abstract

*Ophrys aveyronensis* is an orchid with disjunct geographic distribution. For biogeographic and conservation purpose, we sequenced its complete plastome using Illumina data. The complete plastome is 146,816 bp in length, consisting of a pair of inverted repeats (IRs) of 25,006 bp, a large single-copy (LSC) region and a small single-copy region (SSC) of 80,495 and 16,309 bp, respectively. It was found to contain 133 genes, including 86 protein-coding genes, up to 39 trRNA genes and 8 rRNA genes. The overall GC content of the plastid genome is 36.9%. Phylogenetic inference confirms that *O. aveyronensis* is very close to *O. sphegodes*.

*Ophrys sphegodes* Mill. subsp. *aveyronensis* (Wood 1983) or *Ophrys aveyronensis* (Delforge 1984) is an orchid species that was first described as endemic to a geographically restricted area in the ‘Grands Causses’ region (Southern France). Ever since, phenotypically similar populations were discovered sporadically, in North-Eastern Spain (Hermosilla and Soca 1999). These Iberian populations are considered as a distinct taxon by some authors (*i.e. O. vitorica*, Kreutz 2007; Delforge 2016). However, the level of genetic differentiation between the two allopatric groups has never been assessed.

We used a CTAB 2X protocol to extract genomic DNA from an individual of *Ophrys aveyronensis* collected near Lapanouse-de-Cernon, France (N 43.98945° E 3.09045°) with appropriate permit and deposited in the collection of the University of Perpignan Via Domitia under accession number 18-GS-049. Whole genomic libraries were prepared and sequenced in paired-end mode (2 × 150 bp) using Illumina technology by Novogene Co., Ltd (HK). We used NOVOPlasty v.2.7.2 (Dierckxsens et al. 2017) to reconstruct chloroplast genome, the web-based interface of GeSeq v.167 (Tillich et al. 2017) to carry on gene annotation, as well as the viewing and editing features of Geneious v.11.0.5 (https://www.geneious.com). The sequence is available from GenBank (Accession no.: MN120441).

The plastid genome of *O. aveyronensis* is a circular molecule of 146,816 bp in length, comprising a large single-copy (LSC) region and a small single-copy region (SSC) of 80,495 and 16,309 bp, respectively, separated by two inverted repeat regions (IR) of 25,006 bp (Figure 1). We annotated 110 distinct genes, including 79 protein-coding genes, 4 ribosomal RNA genes (all located in the IR) and (up to) 27 distinct tRNA genes. The genome contained 90 unique genes, 19 genes duplicated in the IRs and 1 (*trn*M-CAU) triplicated in the LSC. Among annotated genes, 6 contained one intron (*atp*F, *ndh*A, *ndh*B, *rpl*2, *rpo*C1, *rps*12, *rps*16) and two contained two introns (*clp*P and *ycf*3). The overall GC content of 39.6% was of 34.3, 29.4 and 43.5% in the LSC, SSC and IR regions respectively (see Supplementary materials for more details).

**Figure 1.**
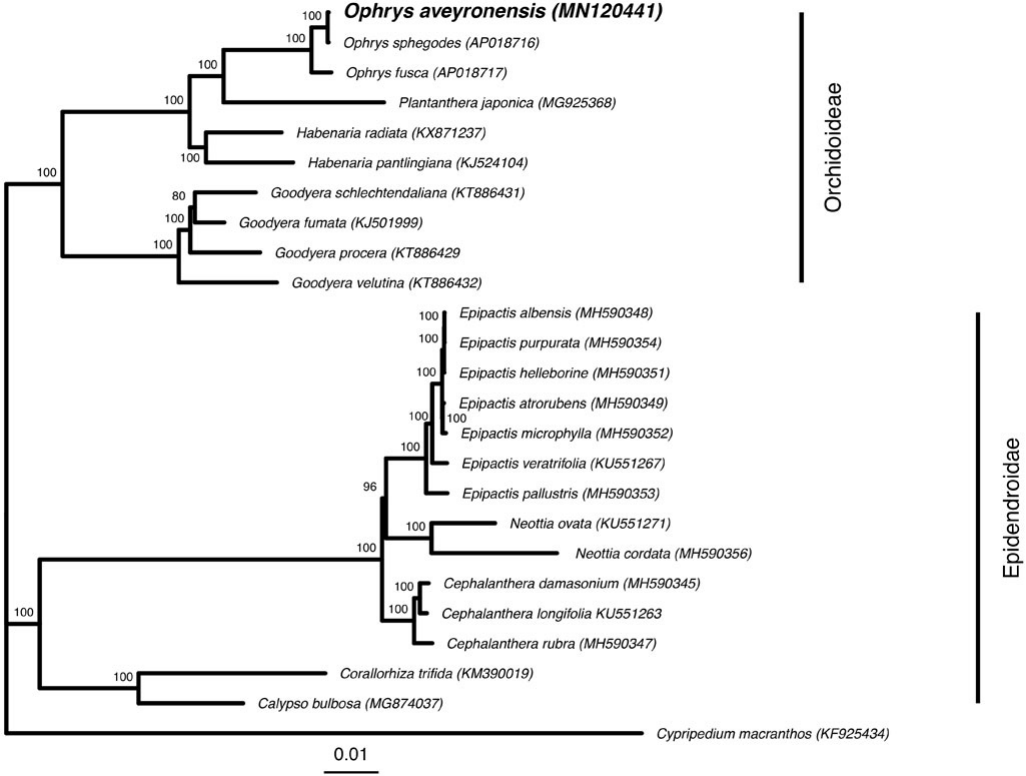
Phylogenetic position of *Ophrys aveyronensis* inferred by Maximum Likelihood method based on 25 whole-plastome orchid sequences for which genera occur in the Euro-Mediterranean region. Node supports correspond to bootstrap values.

The *Ophrys aveyronensis* plastid genome was comparable in size and structure to other published plastomes of photosynthetic orchids. We used MAFFT v7.3.88 (Katoh et al. 2002; Katoh and Standley 2013) to align the plastome of *O. aveyronensis* with a set of other previously published plastid genomes and reconstructed a phylogenetic tree to verify its systematics placement with RAxML v.8.2.11 (Stamatakis 2014). The phylogenetic hypothesis we generated confirms the strong similarity of *Ophrys aveyronensis* plastid genome with the one of *Ophrys sphegodes* (99.8%) and to a lesser extent to the one of *Ophrys fusca iricolor* (96.2%) recently published by Roma et al. (2018).

## Supplementary Material

Supplemental Material
