# Supplementary material for: Characterization of the complete plastome of Ophrys aveyronensis, a Euro-Mediterranean orchid with an intriguing disjunct geographic distribution
Source: Mitochondrial DNA B Resour. 2019 Sep 26;4(2):3256–7. doi: 10.1080/23802359.2019.1670748 (PMC7710315; doi:10.1080/23802359.2019.1670748)
Supplement: Supplemental Material [file TMDN_A_1670748_SM2497.zip › Supplementary_materials.pdf]

Supplementary online material to 'Characterization of the complete plastome of *Ophrys aveyronensis*, a Euro-Mediterranean orchid with an intriguing disjunct geographic distribution' (Bertrand et al.)

DNA extraction and sequencing

We used a CTAB2X protocol to extract genomic DNA from a specimen of *Ophrys aveyronensis* (collected near 'Lapanouse-de-Cernon, Aveyron, France'; (N 43.98945° E 3.09045°) on 12-05-2018; under permit 'Arrêté préfectoral n°2018-s-20' issued by the 'Direction Régionale de l'Environnement de l'Aménagement et du Logement (DREAL)' from the 'Région 'Occitanie', on 11-june-2018). Whole genomic libraries were prepared and sequenced in paired-end mode (2 x 150 bp, insert size: 350 bp) using Illumina technology by Novogene Co., Ltd (HK).

Plastid genome reconstruction

We obtained 319,740,401 of raw reads (95.9 Go) of which 10,575,367 of Read 1 (3.3%) and 7,634,971 (2.4%) of Read 2 were found to contain adapters by cutadapt v1.8 (Martin, 2011). For plastid genome assembly, we used as seed the only plastid sequence of *Ophrys aveyronensis* currently available from GenBank: a partial sequence of a tRNA-Leu (trnL) gene (Accession AY014557, Soliva et al. 2001). We reconstructed plastid genome with NOVOplasty v2.7.2 (Dierckxsens et al. 2016) with the parameter 'Max memory' set to 15. This resulted in subsample of 24,531,544 input reads (3.84% of the total raw reads). All other parameters were kept to default/and or standard values for 150 bp Illumina reads and an insert size of 350 bp. We run the analyses i) with, and ii) without, the reference provided by the recently published plastid genome of *Ophrys sphegodes* (Accession AP018717, Roma et al. 2018). The software aligned 547,644 and 554,068 reads, assembled 294,768 and 312,008 reads for an average organelle coverage of 560X and 566X with the first and second methods, respectively (2.23 and 2.26% of the total reads were found to be plastid-related). The first method provided a single contigs and successfully circularized it but the second one provided three contigs consistent with two different assembly options.

Gene annotation

Gene annotation was performed using the web-based interface of GeSeq v.1.67 (Tillich et al. 2017) and compared to previously published plant genomes thanks to the viewing and editing features of Geneious v.11.0.5 (https://www.geneious.com, see Supplementary Table S1). We used tRNAscan-se v.2.0 and ARAGORN v.1.2.38 (Laslett and Canback 2004) to annotate tRNA genes. The two programs annotated 36 and 39 tRNA genes, respectively. The three following tRNA genes: *trn*K-UUU, *trn*L-UAA and *trn*S-CGA were detected based on ARAGORN annotation only. We noted some incongruences in tRNA gene annotations between the two softwares (e.g. *trn*A-UGC, *trn*D-GUC, *trn*E-UUC, *trn*F-GAA, *trn*G-GCC, *trn*H-GUG, *trn*L-CAA, *trn*M-CAU, *trn*N-GUU, *trn*P-UGG, *trn*R-ACG, *trn*S-UGA, *trn*T-GGU, *trn*Y-GUA). We then drew the gene map of the *O. aveyronensis* plastid genome with OGDRAW v 1.3.1 (Greiner et al. 2019, see Figure S1).

Regarding pseudogenization and gene transfers between organellar and nuclear compartments we confirmed that most of the genes encoding for subunits of the NADH dehydrogenase (*ndh*B, *ndh*C, *ndh*D, *ndh*F, *ndh*G, *ndh*H, *ndh*I, *ndh*J and *ndh*K) were found to be shorter (truncated) compared to other plants. The observed pattern thus displays significant variation even at the intra-generic level, between the three *Ophrys* species as already documented in other plants, including orchids species (e.g. Kim and Chase 2017; Li et al. 2019), and more recently reported in *Ophrys* (Roma et al. 2018).

Supplementary Table S1. List of genes identified in the plastome of *Ophrys aveyronensis*

Gene Category	Group of gene	Name of gene
	Ribosomal RNA genes	*rrn16*[a]; *rrn23*[a]; *rrn4.5*[a]; *rrn5*[a]
Self-replication	Transfer RNA genes	*trn*A-UGC[a], *trn*C-GCA, *trn*D-GUC, *trn*E-UUC [(a)], *trn*F-GAA, *trn*G-GCC, *trn*H-GUG[a], *trn*K-UUU, *trn*L-CAA[a], trnL-UAA, *trn*L-UAG, *trn*M-CAU[b], *trn*N-GUU[a], *trn*P-UGG, *trn*Q-UGG, *trn*R-ACG[a], *trn*R-UCU, *trn*S-CGA, *trn*S-GCU, *trn*S-GGA, *trn*S-UGA, *trn*T-GGU, trnT-UGU, *trn*V-GAC[a], *trn*V-UAC, *trn*W-CCA, *trn*Y-GUA[(a)]
	Small subunit of ribosome	*rps*2, *rps*3, *rps*4, *rps*7[a], *rps*8, *rps*11, *rps*12[a], *rps*14, *rps*15, *rps*16, *rps*18, *rps*19[a]
	Large subunit of ribosome	*rpl*2[a], *rpl*14, *rpl*16, *rpl*20, *rpl*22, *rpl*23[a], *rpl*32, *rpl*33, *rpl*36
	DNA-dependent RNA polymerase	*rpo*A, *rpo*B, *rpo*C1, *rpo*C2
	Subunit of photosystem I (PSI)	*psa*A, *psa*B, *psa*C, *psa*I, *psa*J, *ycf*3, *ycf*4
	Subunit of photosystem II (PSII)	*psb*A, *psb*B, *psb*C, *psb*D, *psb*E, *psb*F, *psb*H, *psb*I, *psb*J, *psb*K, *psb*L, *psb*M, *psb*N, *psb*T, *psb*Z.
Photosynthesis	Subunits of cytochrome b_6f	*pet*A, *pet*B, *pet*D, *pet*G, *pet*L, *pet*N
	Subunits of ATP synthase	*atp*A, *atp*B, *atp*E, *atp*F[c], *atp*H, *atp*I
	Subunits of NADH dehydrogenase	*ndh*A[c], *ndh*B[a,c], *ndh*C, *ndh*D, *ndh*E, *ndh*F, *ndh*G, *ndh*H, *ndh*I, *ndh*J, *ndh*K
	Large subunits of Rubisco	*rbc*L
	Maturase	*mat*K
	Envelope membrane protein	*cem*A
Other genes	Subunit of acetyl-CoA carboxylase	*acc*D
	C-type cytochrome synthesis gene	*ccs*A
	Protease	*clp*P[c]
	Component of TIC complex	*ycf*1
	Translation initiation factor IF-1	*inf*A
Genes of unknown function		*ycf*2[a]

[a] Duplicated (or possibly duplicated) gene (present in the IR regions)
[b] Triplicated (or possibly triplicated) gene
[c] Coding gene containing intron(s)

Phylogenetic inference

To verify the phylogenetic placement of the plastome of *O. aveyronensis* we reconstructed, we first retrieved orchid whole sequences of plastid genomes available from GenBank. We typed the following request *"Orchidaceae"[Organism] AND (biomol_genomic[PROP] AND plastid[filter]) AND "complete genome"* which returned 387 entries (on 2019/04/17). We removed duplicates and accessions explicitly mentioning the terms ‘hybrid’ and/or ‘cultivar’. Out of this initial dataset, we aligned 25 sequences consisting of a set of representatives of species from the genera occurring in the Euro-Mediterranean region using MAFTT v7.3.88 (Katoh et al. 2002; Katoh and Standley 2013) with default settings. We then used RAxML v.8.2.11 (Stamatakis 2014) with a GTR CAT I substitution model, the following arguments: ‘-f a -x 1’ and an automatic bootstrap procedure to assess nodes support.

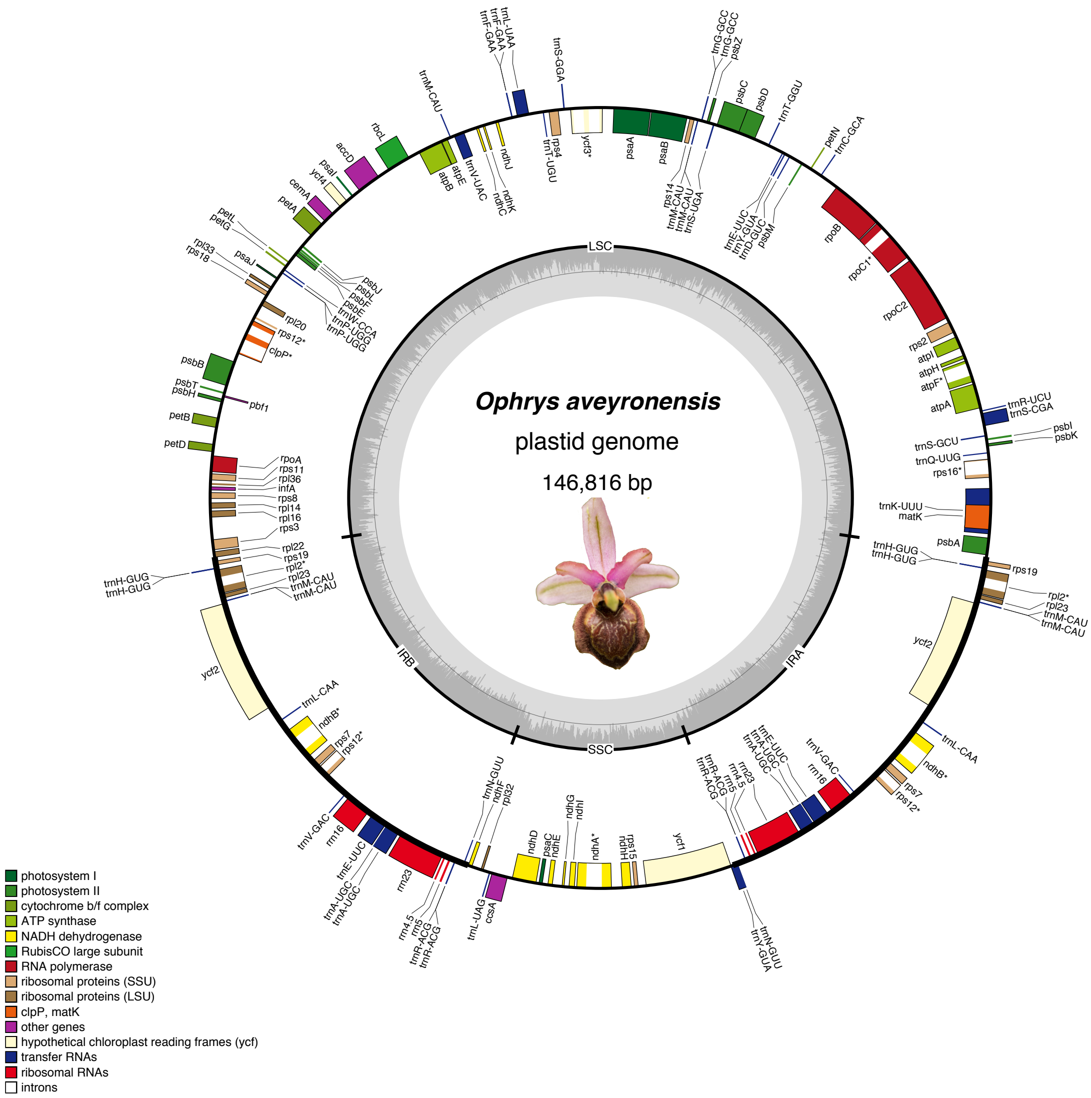

Figure S1 Gene map of *Ophrys aveyronensis* plastid genome. Genes drawn inside the circle are transcribed in the clockwise direction, and genes drawn outside are transcribed in the counter-clockwise direction. Dark and light grey in the inner circle corresponds to GC and AT content, respectively.

References

Dierckxsens N, Mardulyn P, Smits G. 2016. NOVOPlasty: De novo assembly of organelle genomes from whole genome data. Nucleic Acids Res., 45:e18.

Greiner S, Lehwark P, Bock R. 2019. OrganellarGenomeDRAW (OGDRAW) version 1.3.1: expanded toolkit for the graphical visualization of organellar genomes. Nucleic Acids Res: *gkz* 238.

Katoh K, Misawa K, Kuma KI. 2002. MAFFT: a novel method for rapid multiple sequence alignment based on fast Fourrier transform. Nucleic Acids Res., 30:3059-3066.

Katoh K, Standley DM. 2013. MAFFT multiple sequence alignment software version 7: improvements in performance and usability. Mol. Biol. Evol., 30:772-780.

Kim HT, Chase MW. 2017. Independent degradation in genes of the pastid *ndh* gene family in species of the orchid genus *Cymbidium* (Orchidaceae; Epidendroideae). Plos ONE, 12(11): e0187318.

Laslett D, Canback B. 2004. ARAGORN, a program for the detection of transfer RNA and transfer-messenger RNA genes in nucleotide sequences. Nucleic Acids Res*.,* 32:11-16.

Li ZH, Xiao M, De-Yi W, Yun-Xia L Cheng-Wang W, Xia-Hua J. 2019. Evolution of plastid genomes of *Holcoglossum* (Orchidaceae) with recent radiation. BMC Evol. Biol.**,** 19:63.

Lowe TM, Chan PP. 2016. tRNAscan-SE on-line: search and contextual analysis of transfer RNA genes. Nucleic Acids Res., 44:W54-W57.

Martin M. 2011. Cutadapt removes adapter sequences from high-throughput sequencing reads. EMBnet.journal, 17:10-12.

Roma L, Cozzolino S, Schlüter PM, Scopece G, Cafasso D. 2018. The complete plastid genomes of *Ophrys iricolor* and *O. sphegodes* (Orchidaceae) and comparative analyses with other orchids. PLoS ONE, 13:e204174.

Soliva M, Kocyan A, Widmer A. 2001. Molecular phylogenetics of the sexually deceptive orchid genus *Ophrys* (Orchidaceae) based on nuclear and chloroplast DNA sequences. Mol. Phyl. Evol., 20:78-88.

Stamatakis A. 2014. RAxML version 8: a tool for phylogenetic analysis and post-analysis of large phylogenies. Bioinformatics, 30:1312-1313.

Tillich M, Lehwark P, Pellizzer T, Ulbricht-Jones ES, Fischer A, Bock R, Greiner S. 2017. GeSeq – versatile and accurate annotation of organelle genomes. Nucleic Acids Res. 45:W6-W11.
